# Efficacy and cost-effectiveness of VATS versus chest tube drainage in first-episode primary spontaneous pneumothorax with blebs: a propensity score-matched retrospective study

**DOI:** 10.1186/s12890-026-04155-9

**Published:** 2026-02-05

**Authors:** Qingcai Lin

**Affiliations:** Department of Thoracic Surgery (Q.C. Lin.), Longyan First Affiliated Hospital of Fujian Medical University, Longyan, Fujian China

**Keywords:** Primary spontaneous pneumothorax, Video-assisted thoracoscopic surgery, Chest tube drainage, Cost-effectiveness analysis, Propensity score matching

## Abstract

**Background:**

Optimal management for first-episode primary spontaneous pneumothorax (PSP) with pulmonary blebs remains uncertain, balancing recurrence prevention against procedural costs. This study compared video-assisted thoracoscopic surgery (VATS) and chest tube drainage in terms of recurrence prevention and cost-effectiveness, incorporating sensitivity analyses to evaluate robustness across variable assumptions.

**Methods:**

In a retrospective cohort (2010–2020), 245 first-episode PSP patients with computed tomography (CT)-confirmed blebs were included. Propensity score matching (1:1, caliper = 0.02) balanced baseline characteristics (age, bleb size, etc.), generating 33 matched pairs. Primary outcomes were recurrence rate and incremental cost-effectiveness ratio (ICER).

**Results:**

VATS reduced 5-year recurrence rates from 48.5% to 12.1% *(P* = 0.004; absolute risk reduction [ARR] = 36.4%, number needed to treat [NNT] = 2.75) and improved recurrence-free interval (hazard ratio [HR] = 0.166, *P* < 0.001). The base-case ICER was ¥160,300 per quality-adjusted life-year (QALY) gained (¥48,937 per recurrence avoided), with 99.14% probability of cost-effectiveness at China’s World Health Organization (WHO)-recommended willingness-to-pay (WTP) threshold (¥287,247 / QALY). Sensitivity analyses confirmed robustness: ICER remained favorable at ¥156,338 / QALY when pneumothorax utility dropped to 0.5; a 20% cost increase yielded ¥192,200 / QALY. Discount rate variations (3%: ¥159,800 / QALY; 6%: ¥138,029 / QALY) maintained > 98.4% cost-effectiveness probability.

**Conclusions:**

VATS reduces recurrence by 83.4% in first-episode PSP with blebs and demonstrates robust cost-effectiveness. Early surgical intervention should be considered for patients with blebs across diverse resource settings.

**Supplementary Information:**

The online version contains supplementary material available at 10.1186/s12890-026-04155-9.

## Background

Primary spontaneous pneumothorax (PSP), defined as the presence of air in the pleural cavity in the absence of traumatic injury, most commonly occurs in young adults [1]. Its reported incidence varies between 15.5 and 22.7 cases per 100,000 population, with a consistently observed male predominance reflected in a female-to-male ratio of 1:3.3 to 1:5 [2, 3]. Up to 40% of first-episode patients experience recurrence within 5 years, particularly those with pulmonary blebs visualized on computed tomography (CT) imaging [4–7]. While current international guidelines recommend chest tube drainage as initial management for primary episodes, growing evidence suggests this approach may be suboptimal given high recurrence rates and associated long-term healthcare burdens [8–10]. 

Video-assisted thoracoscopic surgery (VATS) has demonstrated superior efficacy in recurrent PSP, with randomized trials reporting recurrence rates below 5% [11–13]. However, its role in first-episode PSP remains contentious due to concerns over procedural costs and potential overtreatment. Recent economic analyses reveal that recurrence-related expenses, including emergency visits, repeated hospitalizations, and productivity loss, may increase total costs compared to initial surgical intervention [14–17]. Although the 2025 cost-utility model proposed that VATS could be cost-effective, there was still a significant evidence gap for comparison among patients with the same underlying conditions and bulla characteristics [18]. Existing studies predominantly focus on recurrent PSP populations, lacking robust data on first-episode cohorts with radiologically confirmed blebs [16, 17]. Furthermore, previous comparisons are compromised by selection bias from unadjusted baseline imbalances in critical prognostic factors such as bleb size and age [10]. Crucially, no real-world studies have employed rigorous propensity score matching (PSM) to evaluate both clinical outcomes and cost-effectiveness in this population.

To address these limitations, this retrospective cohort study applies 1:1 PSM methodology to minimize confounding variables while comparing VATS and chest tube drainage in first-episode PSP patients with CT-confirmed blebs. We assess not only recurrence outcomes but also incremental cost-effectiveness ratios (ICERs), utilizing World Health Organization (WHO)-recommended willingness-to-pay (WTP) thresholds tailored for emerging economies [19, 20]. 

## Methods

### Study design and data source

This study utilized electronic medical records from the Hospital Information System of Longyan First Affiliated Hospital of Fujian Medical University from January 2010 to May 2020. We identified patients with first-episode PSP and radiologically confirmed ruptured pulmonary blebs (subpleural air cysts with pleural discontinuity on chest CT). For the purposes of this study, the term “blebs” encompasses subpleural air cysts of varying sizes, including those traditionally classified as bullae (> 2 cm), as all included patients had no underlying lung disease, thus maintaining the classification as primary spontaneous pneumothorax (PSP). The cohort was stratified by initial treatment into two groups: (1) VATS group undergoing video-assisted thoracoscopic bullectomy with pleurodesis, and (2) chest tube group receiving closed-tube thoracostomy as intervention. Ethical approval was obtained from the Institutional Review Board (Approval No.: LYREC2025-K165-01) with waiver of informed consent for anonymized data analysis.

### Participant selection

Eligibility criteria required: (a) definitive diagnosis of first-episode PSP with blebs rupture necessitating intervention, (b) absence of underlying cardiopulmonary diseases, and (c) complete medical records including operative notes, itemized cost statements, and a 60-month follow-up. Exclusion criteria systematically eliminated patients with: (i) secondary pneumothorax (trauma, chronic obstructive pulmonary disease [COPD] and cystic fibrosis), (ii) prior ipsilateral thoracic surgery, (iii) > 20% missing key variables, or (iv) concurrent major procedures (e.g., lobectomy). Initial screening yielded 486 cases, with 245 meeting inclusion criteria (VATS group = 188, Chest tube group = 57) prior to matching (see Appendix 1).

### Propensity score matching

To address baseline imbalances, propensity scores were estimated using logistic regression with the following covariates: age, sex, smoking status, laterality of pneumothorax, maximum bleb diameter (categorized as < 1 cm, 1–5 cm, > 5 cm), number of blebs (1–2, 3–5, > 5), and degree of lung collapse (see Appendix 2). One-to-one nearest-neighbor matching was performed with a caliper width of 0.02 standard deviations of the propensity score logit, resulting in 33 well-balanced pairs. Standardized mean differences (SMD) decreased from 0.631 (pre-match) to 0.081 (post-match), confirming adequate balance (SMD < 0.1 considered negligible).

### Outcome measures

The study evaluated two endpoints through comprehensive follow-up and economic analysis. The first outcome was ipsilateral pneumothorax recurrence within 5 years post-intervention, rigorously defined as radiographically confirmed pneumothorax. For the second outcome, we conducted a formal cost-effectiveness analysis from the healthcare payer perspective, calculating incremental cost per quality-adjusted life-year (QALY) gained.

### Statistical analysis

Continuous variables were reported as medians with interquartile ranges (IQR) and compared using Mann-Whitney U tests (pre-match), Wilcoxon signed-rank tests (post-match) and paired t-test (post-match). Categorical variables were analyzed with *χ²* or Fisher’s exact tests as appropriate, transitioning to McNemar’s test for matched pairs. Time-to-event outcomes were evaluated through Kaplan-Meier survival curves with log-rank testing and Cox proportional hazards regression adjusted for residual confounders.

We conducted a cost-effectiveness analysis from the healthcare payer perspective, adopting the WHO-CHOICE framework [19]. Incremental cost-effectiveness ratios (ICERs) as: ICER = ΔCost/ΔEffect (QALYs gained; recurrence avoided), were evaluated against China’s WHO-recommended WTP thresholds (1–3× gross domestic product [GDP] per capita, ¥95,749–¥287,247/QALY) [21]. Probabilistic sensitivity analysis incorporated Monte Carlo simulations (*n* = 10,000) with parameters calibrated to regional cost databases (Appendix 3). All analyses were conducted using the Social Sciences (SPSS) version 29.0 (International Business Machines [IBM] Corp, Armonk, NY) with *P* < 0.05 defining statistical significance.

### Sample size considerations

The sample size calculation was performed a priori using the two-independent proportions formula, based on preliminary institutional data demonstrating anticipated recurrence rates of 45.6% in the chest tube group versus 11.7% in the VATS group. With *α* = 0.05 (two-tailed) and *β* = 0.20 (80% power), the initial estimation yielded a requirement of 26 patients per group. To account for potential limitations inherent to retrospective studies, including approximately 5% anticipated data incompleteness from missing medical records, 10% expected loss to follow-up in longitudinal outcomes assessment, and 5% reduction in matching efficiency during propensity score analysis, we conservatively inflated the sample size by 20%. This adjustment established a minimum target of 32 patients per treatment arm before matching. The final propensity-matched cohort successfully achieved 33 pairs (*n* = 66), providing enhanced statistical robustness. Post-hoc analysis confirmed this sample size afforded 86.2% power to detect the observed clinically significant hazard ratio (HR) for recurrence, while maintaining adequate precision for cost-effectiveness evaluations.

## Results

The propensity score-matched cohort analysis included 33 patient pairs (*n* = 66) with well-balanced baseline characteristics after matching (mean SMD = 0.081)(Table 1). The VATS group and chest tube group showed comparable distributions of age (median 50 vs. 46 years, *P* = 0.341), sex (84.8% male in both), smoking status (54.5% vs. 66.7%, *P* = 0.125), and bleb characteristics (large blebs > 5 cm: 6.1% in both groups). No significant differences existed in complication rates (36.4% vs. 39.4%, *P* = 1.000) or degree of lung collapse (> 50% collapse: 63.6% vs. 57.6%, *P* = 0.754).

**Table 1:** Baseline characteristics before and after propensity score matching

Variables	Before Matching		After Matching		Statistical Analysis	SMD
	Chest Tube (*n* = 57)	VATS (*n* = 188)	Chest Tube (*n* = 33)	VATS (*n* = 33)	Pre-Match Test	Pre-Match vs. Post-Match
					Post-Match Test	
Categorical Variables						
Sex						
Male	46(80.7%)	161(85.6%)	28(84.8%)	28(84.8%)	*P* = 0.404	0.131 vs. 0
Female	11(19.3%)	27(14.4%)	5(15.2%)	5(15.2%)	*P* = 1.000	
Age						
<45 years old	21(36.8%)	161(85.6%)	16(48.5%)	16(48.5%)	*P* < 0.001	0.725 vs. 0
≥45 years old	36(63.2%)	27(14.4%)	17(51.5%)	17(51.5%)	*P* = 1.000	
Smoking state						
No	23(40.4%)	136(72.3%)	11(33.3%)	15(45.5%)	*P* < 0.001	0.679 vs. 0.252
Yes	34(59.6%)	52(27.7%)	22(66.7%)	18(54.5%)	*P* = 0.125	
The laterality of PSP						
Left	22(38.6%)	83(44.1%)	13(39.4%)	17(51.5%)	*P* = 0.542	0.112 vs. 0.245
Right	35(61.4%)	105(55.9%)	20(60.6%)	16(48.5%)	*P* = 0.453	
Degree of lung collapse volume						
30%-50%	26(45.6%)	93(49.5%)	14(42.4%)	12(36.4%)	*P* = 0.652	0.078 vs. 0.123
>50%	31(54.4%)	95(50.5%)	19(57.6%)	21(63.6%)	*P* = 0.754	
Maximum blebs diameter						
<1 cm	10(17.5%)	127(67.6%)	8(24.2%)	10(30.3%)	*P* < 0.001	1.175 vs. 0.137
1–5 cm	36(63.2%)	59(31.4%)	23(69.7%)	21(63.6%)	*P* = 0.157	0.672 vs. 0.130
>5 cm	11(19.3%)	2(1.1%)	2(6.1%)	2(6.1%)		0.631 vs. 0
Number of blebs						
1–2	19(33.3%)	163(86.7%)	16(48.5%)	16(48.5%)	*P* < 0.001	1.300 vs. 0
3–5	21(36.8%)	20(10.6%)	13(39.4%)	13(39.4%)	*P* = 1.000	0.648 vs. 0
>5	17(29.8%)	5(2.7%)	4(12.1%)	4(12.1%)		0.790 vs. 0
Complications (Detailed)						
Any Complication	20(35.1%)	47(25.0%)	13(39.4%)	12(36.4%)	*P* = 0.174	-
Prolonged air leak (> 5 days)	6(10.5%)	20(10.6%)	5(15.2%)	4(12.1%)		
Subcutaneous Emphysema	9(15.8%)	8(4.3%)	6(18.2%)	1(3.0%)		
Atelectasis	4(7.0%)	10(5.3%)	2(6.1%)	2(6.1%)		
Surgical site infection	5(8.8%)	6(3.2%)	3(9.1%)	2(6.1%)		
Fever requiring antibiotics	15(26.4%)	26(13.8%)	9(27.3%)	9(27.3%)		
No complications	37(64.9%)	141(75.0%)	20(60.6%)	21(63.6%)	*P* = 1.000	
Status of recurrence						
Recurrence	26(45.6%)	22(11.7%)	16(48.5%)	4(12.1%)	*P* < 0.001	-
No recurrence	31(54.4%)	166(88.3%)	17(51.5%)	29(87.9%)	*P* = 0.004	
Continuous Variables						
Age (years)	53(41–62)	28(21–37)	50(28.5–59.5)	46(26-57.5)	*P* < 0.001	-
					*P* = 0.341	
Length of hospital stay (days)	7(4.5–10.5)	8(6–10)	7(4-10.5)	8(7-10.5)	*P* = 0.022	-
					*P* = 0.042	
Time to recurrence (months)	15(2–60)	60(60–60)	15(2–60)	60(60–60)	*P* < 0.001	-
					*P* < 0.001	
Total cost(¥)	6,218(3,791-9,100)	25,247(20,656 − 28,766)	6,256(3,769-9,306)	26,922(23,455 − 33,206)	*P* < 0.001	-
					*P* < 0.001	

Data presented as n (%) for categorical variables and median (IQR) for continuous variables

Propensity score matching (1:1, caliper = 0.02) achieved negligible imbalance (mean SMD: 0.631→0.081). Pre-matching p-values from Chi-square test, Fisher’s exact test and Mann-Whitney U test. Post-matching p-values from McNemar test, Bowker’s test, Paired t-test and Wilcoxon signed-rank test. The PSM algorithm and the baseline characteristics before and after matching are detailed in Appendix 1

*Abbreviations*: *IQR* Interquartile range, *PSP* Primary spontaneous pneumothorax, *SMD* Standardized mean difference, *VATS* Video-assisted thoracoscopic surgery

**Fig. 1:**
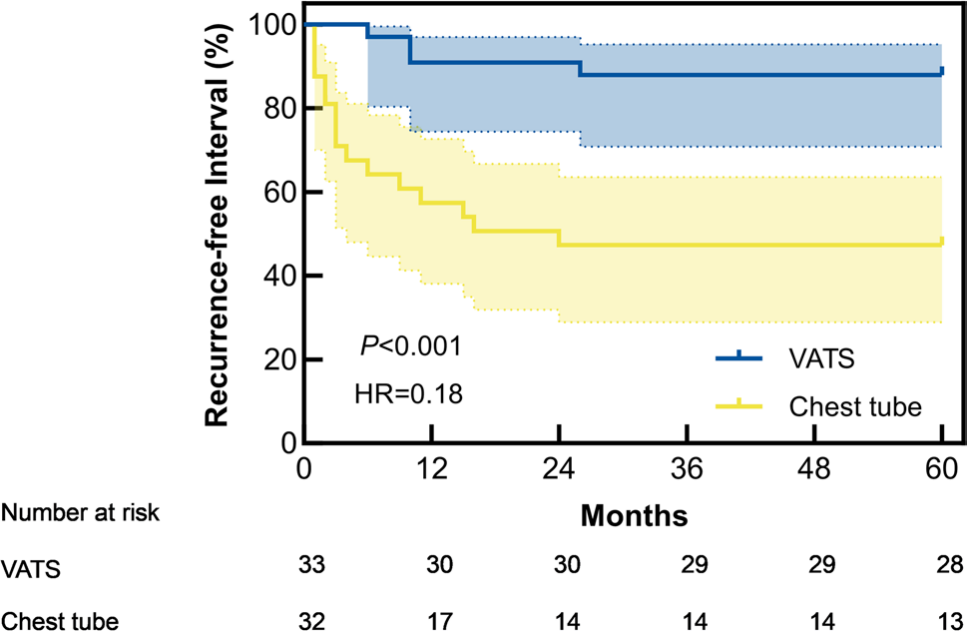
Kaplan-Meier curve for recurrence-free interval. Chest tube group: median 24 months (95% CI: 21.97–41.38). VATS group: not reached (95% CI: 48.99–59.62). Log-rank test: *χ²* = 13.464, *P* < 0.001

VATS demonstrated superior efficacy in preventing pneumothorax recurrence. The recurrence rate was significantly lower in the VATS group (12.1%, 4/33) compared to the chest tube group (48.5%, 16/33), with an absolute risk reduction (AAR) of 36.4% (*P* < 0.001). This corresponds to a number needed to treat of 2.75, indicating that for every 3 patients treated with VATS instead of chest tube drainage, one additional recurrence would be prevented. Time-to-event analysis revealed markedly prolonged recurrence-free interval in the VATS group (median not reached, 95% confidence interval[CI]:48.99–59.62 months) versus the chest tube group (median 24 months, 95% CI:21.97–41.38 months; log-rank *P* < 0.001)(Fig. 1). Cox proportional hazards analysis confirmed VATS independently reduced recurrence risk by 83.4% (*HR* = 0.166, 95% CI:0.055–0.498, *P* < 0.001).

Regarding complications, as detailed in Table 1, the most common complications included prolonged air leak (PAL) (> 5 days), subcutaneous emphysema, atelectasis, surgical site infection (in the VATS group), and fever requiring antibiotics. There was no significant difference in the overall complication rate between the two matched groups (*P* = 1.000). Notably, PAL was observed in 5 patients (15.2%) in the chest tube group, three of whom subsequently required crossover to VATS as a rescue therapy for the ongoing air leak. These patients remained in the chest tube group for the intention-to-treat analysis of recurrence. The costs associated with this crossover surgery were included in the economic evaluation of the chest tube strategy.

The median length of hospital stay was longer in the VATS group (8 days) than in the chest tube group (7 days) (*P* = 0.042). This difference is likely attributable to the postoperative recovery period following general anesthesia and the standard institutional protocol for monitoring chest tube drainage after VATS. In the chest tube group, prolonged stays were often associated with PAL or the management of recurrence.

The economic evaluation demonstrated that while VATS incurred higher initial costs (median ¥26,922, IQR 23,455 − 33,206 vs. ¥6,256, IQR 3,769-9,306; *P* < 0.001), it showed favorable cost-effectiveness. The ICER was ¥160,300 per QALY gained (or ¥48,937 per recurrence avoided). This calculation incorporated utility values derived from the published literature, with an assumed 14-day recurrence duration (Table 2) [9, 18, 22]. This resulted in a total QALY gain of 3.67 with VATS. The ICER remained substantially below China’s WHO-recommended WTP threshold of ¥287,247 per QALY gained (3×GDP per capita).

**Table 2:** Cost-effectiveness analysis of VATS versus chest tube

Metric	Value
Incremental Cost (ΔCost)	¥17,813 per patient
Incremental Effect (ΔEffect)	36.4% recurrences avoided
ICER	¥160,300 per QALY gained (or ¥48,937 per recurrence avoided)
WHO Threshold	¥ 287,247 per QALY gained (3×GDP)
Conclusion	VATS cost-effective

WHO threshold = 3×GDP per capita (¥287,247 / QALY in China). Utilities: Stable: 1.0; Pneumothorax: 0.7; Post-recovery: 0.95; Recurrence duration: 14 days. Utility values were sourced from published cost-utility studies and the WHO-CHOICE framework, as direct patient-reported quality-of-life measures were not available in this retrospective study

*Abbreviations*: *GDP* Gross domestic product, *ICER* Incremental cost-effectiveness ratio, *QALY* Quality-adjusted life-year, *WHO* World Health Organization

Sensitivity analyses confirmed the robustness of these findings across varying assumptions (Table 3). When pneumothorax utility values were decreased to 0.5, the ICER remained favorable at ¥156,338 per QALY gained. Even with a 20% increase in procedure costs (¥705,395 total), the ICER of ¥192,200 per QALY gained stayed below the WHO threshold. At a 3% discount rate, the ICER was ¥159,800 per QALY gained with a cost-effectiveness acceptability probability of 98.49%. At a 6% discount rate, the ICER decreased to ¥138,029 per QALY gained while maintaining high cost-effectiveness acceptability (98.47%).

**Table 3:** Sensitivity analysis of cost-effectiveness

Parameter Varied	Scenario	ΔCost (¥)	ΔQALY	ICER (¥ / QALY)	CE Probability at WTP Thresholds
Base Case	Reference	587,829	3.67	160,300	0.9914
Pneumothorax utility	Reduced to 0.5	587,829	3.76	156,338	0.9881
Pneumothorax utility	Increased to 0.8	587,829	3.62	162,384	0.9807
Recurrence Duration	Shortened (7 days)	587,829	3.60	163,286	0.9806
Recurrence Duration	Extended (21 days)	587,829	3.74	157,200	0.9856
Cost variation	+ 20%	705,395	3.67	192,200	0.9322
Cost variation	-20%	470,263	3.67	128,100	0.9981
Discount Rate	0%	587,091	3.67	159,970	0.9873
Discount Rate	3%	506,565	3.17	159,800	0.9849
Discount Rate	6%	587,076	3.67	138,029	0.9847

*Abbreviations*: *CE* Cost-effectiveness, *WTP* Willingness-to-pay

Fig. 1 Kaplan-Meier Curve for Recurrence-Free Interval. Chest tube group: median 24 months (95% CI: 21.97-41.38). VATS group: not reached (95% CI: 48.99-59.62). Log-rank test: χ² = 13.464, P < 0.001

**Fig. 2:**
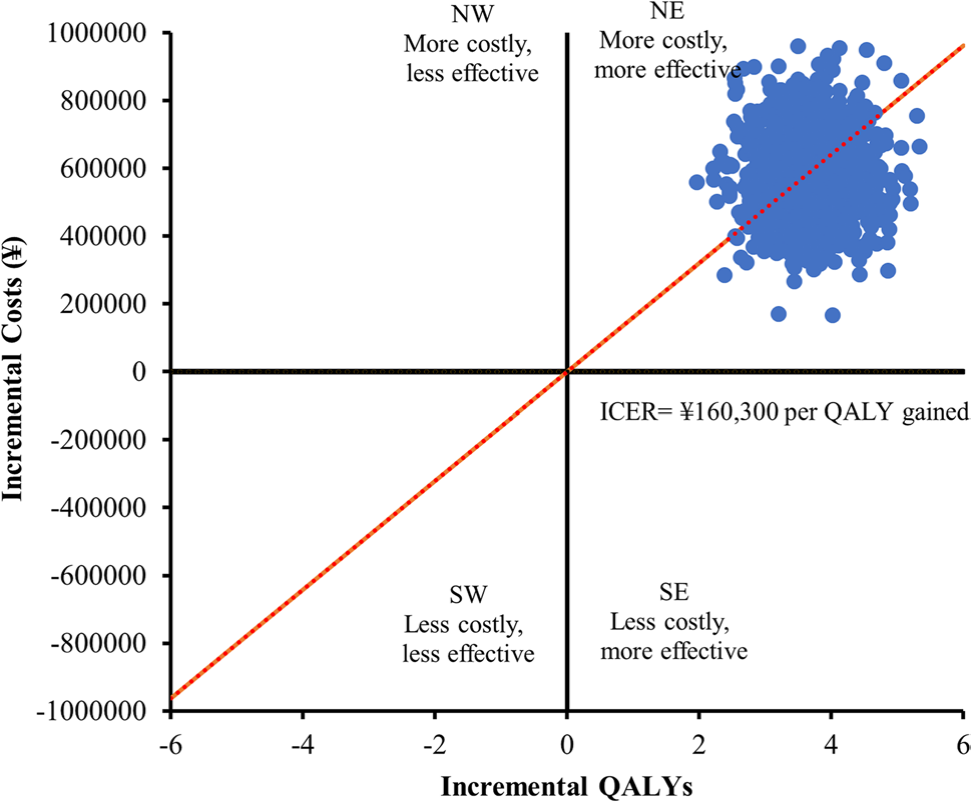
Cost-effectiveness plane for VATS versus chest tube

Fig. 2 Cost-Effectiveness Plane for VATS versus Chest Tube

**Fig. 3:**
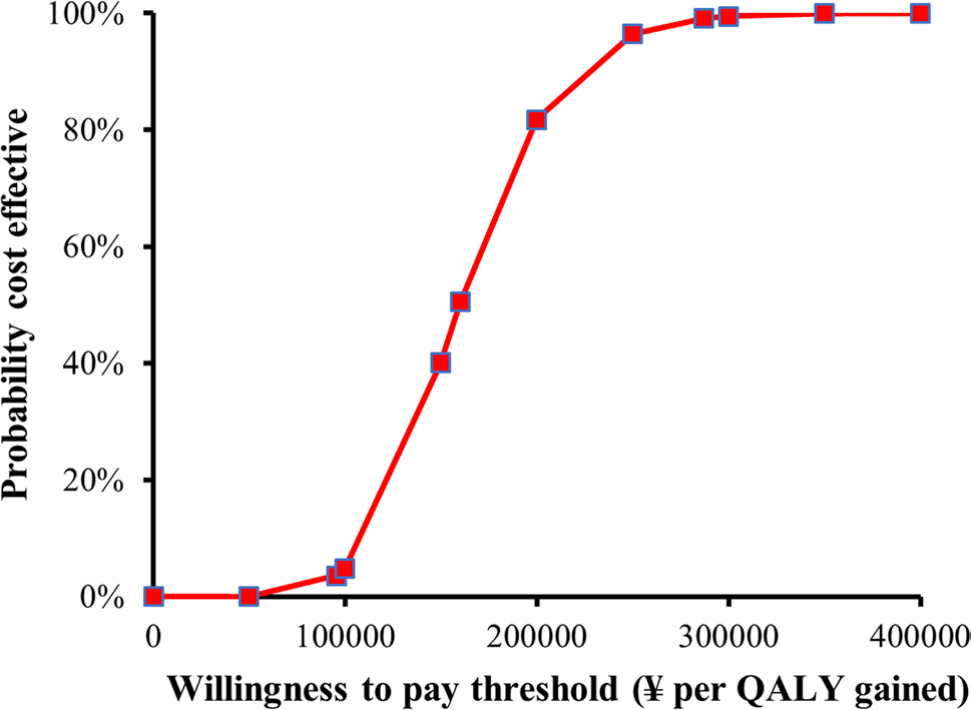
Cost-effectiveness acceptability curve. Probability of VATS being cost-effective: 99.14% at WHO threshold (3×GDP, ¥287,247 / QALY) vs. 3.57% at lower threshold (1×GDP, ¥95,749 / QALY)

Fig.3 Cost-Effectiveness Acceptability Curve. Probability of VATS being cost-effective: 99.14% at WHO threshold (3×GDP, ¥287,247 / QALY) vs. 3.57% at lower threshold (1×GDP, ¥95,749 / QALY)

The Monte Carlo simulation (*n* = 10,000 iterations) demonstrated consistent cost-effectiveness outcomes, with 100.0% of iterations in the cost-effectiveness plane (Fig. 2) falling within the northeast quadrant (higher effectiveness at higher cost), centered at an incremental cost of ¥587,829 and 3.67 QALYs gained. The acceptability curve analysis (Fig. 3) revealed a 99.14% probability of VATS being cost-effective at China’s WHO-recommended WTP threshold of ¥287,247 / QALY (3×GDP per capita), decreasing to 3.57% probability at the lower 1×GDP threshold of ¥95,749 / QALY.

Post-hoc analysis confirmed adequate statistical power (86.2%) for detecting the observed treatment effect. Hospital stay duration showed a clinically meaningful difference between groups (median 8 days, IQR 7-10.5 vs. 7 days, IQR 4-10.5; *P* = 0.042). The comprehensive matching protocol and extensive sensitivity analyses address potential limitations related to the retrospective design while reinforcing the validity of our findings regarding both clinical outcomes and cost-effectiveness.

## Discussion

This propensity score-matched study demonstrates that VATS significantly reduces recurrence risk in first-episode PSP patients with pulmonary blebs while achieving robust cost-effectiveness. Our findings reveal a 36.4% absolute risk reduction (ARR) with VATS compared to chest tube drainage, translating to one recurrence prevented per three patients treated, alongside an 83.4% hazard reduction (*HR* = 0.166) over 5 years. These results challenge current guideline recommendations favoring conservative management for initial episodes and provide evidence to support early surgical intervention in selected patients [8, 9, 23]. 

We acknowledge the reviewer’s point regarding the terminology of blebs versus bullae. In this study, we included patients with subpleural air cysts detected on CT, and used the term “blebs” broadly, consistent with several contemporary studies on PSP [5, 24]. Importantly, all patients had no clinical or radiological evidence of underlying lung disease (e.g., COPD), which maintains their classification as having primary spontaneous pneumothorax according to international definitions [4]. The presence of larger air cysts (> 5 cm) in a minority of our PSP patients highlights the pathological continuum and the potential for more severe structural abnormalities even in the absence of classic secondary causes.

The clinical superiority of VATS aligns with emerging pathophysiological understanding of PSP. Large (> 5 cm) and numerous blebs, validated as independent recurrence predictors in contemporary studies, create structural vulnerabilities that chest tube drainage fails to address [12, 25, 26]. VATS directly eliminates these substrates through bleb resection while pleural abrasion induces adhesions, reducing recurrence risk more effectively than reactive pleural inflammation from drainage alone [13, 27]. Our data confirm that patients with radiologically confirmed blebs constitute a high-risk subgroup warranting aggressive initial management, particularly given the 48.5% recurrence rate observed in conservatively managed patients. The high recurrence rate observed in our conservatively managed cohort (48.5%) underscores that patients with radiologically confirmed blebs represent a high-risk subgroup, a risk potentially compounded by the older age profile in our study.

Furthermore, the occurrence of PAL in 15.2% of the chest tube group, often necessitating surgical intervention, underscores another limitation of conservative management. VATS not only prevents future recurrences but also addresses the acute complication of PAL more definitively.

From a health economics perspective, VATS demonstrates compelling value despite higher upfront costs. The ICER of ¥160,300 per QALY gained falls substantially below China’s WHO-recommended WTP threshold (¥287,247 / QALY), with Monte Carlo simulations (*n* = 10,000) confirming a 99.14% probability of cost-effectiveness. This economic viability persists even under conservative assumptions, including a 20% cost increase (ICER ¥192,200 / QALY), and addresses critical gaps in guideline development for resource-constrained settings.

There exists an inverse relationship between discount rates and ICERs, whereby higher discount rates (6%) yield lower ICERs compared to lower rates (3%). This observation aligns with established principles in health economics. The majority of intervention costs are incurred at the outset (e.g., surgical procedures), making these costs relatively less sensitive to discounting. Health benefits are realized over the course of patients’ lifetimes; consequently, higher discount rates significantly reduce the present value of quality-adjusted life years. It is important to note that the cost-effectiveness acceptability probability remained highly consistent (> 98.4%) across all scenarios involving varying discount rates. This stability underscores the robustness of the intervention’s value proposition under different discount rate assumptions within policy-relevant ranges. Furthermore, the utilization of real-world cost data in this analysis incorporate actual costs associated with disease recurrence across the treatment continuum with 1:1 PSM matching of basic patient information and bulla characteristics, thereby addressing a key limitation of previous model-based studies (individual differences) [14, 18]. 

The 36.4% ARR provides particularly valuable information for shared decision-making. While relative risk metrics (83.4%) illustrate biological efficacy, ARR quantifies tangible clinical impact in patient-centric terms: without intervention, approximately half of first-episode patients with blebs will experience recurrence within five years; VATS reduces this risk by over one-third [28]. This intuitive metric facilitates risk-benefit discussions with young patients weighing immediate recovery against long-term recurrence prevention.

Methodologically, this study advances PSP research through rigorous propensity score matching that balanced critical prognostic variables, including bleb size distribution and smoking status, achieving near-perfect covariate balance (mean SMD = 0.081). This design minimizes selection bias inherent in retrospective comparisons and responds to calls for higher-quality real-world evidence in pneumothorax management. Our incorporation of itemized 10-year cost data further strengthens economic conclusions by capturing true resource utilization patterns beyond theoretical models.

Several limitations warrant consideration. The single-center retrospective design risks unmeasured confounding despite meticulous matching. Generalizability may be limited to similar healthcare economies. Furthermore, our requirement for CT-confirmed blebs as an inclusion criterion, while strengthening internal validity by ensuring a homogeneous study population, may not reflect all real-world settings where preoperative CT is not universally performed, potentially limiting generalizability. The 5-year follow-up might miss late recurrences beyond this window. Finally, our QALY estimates derived from population utility weights rather than direct patient measures, though extensive sensitivity analyses mitigate this concern. Additionally, the median age of our matched cohort (46–50 years) is higher than the typical peak for PSP (20–35 years). This may reflect our local patient demographics and smoking patterns, and suggests our findings may be particularly relevant for older PSP patients or smokers without overt COPD, potentially limiting generalizability to the youngest PSP populations. This older age profile might also contribute to the observed recurrence rates being higher than those reported in some studies predominantly involving younger patients, as age itself can be a factor in tissue healing and recurrence risk. Future studies should aim to validate these findings in a broader age range.

These findings translate to actionable practice recommendations: prioritizing VATS for first-episode PSP patients exhibiting large (> 5 cm) or numerous blebs on CT imaging represents the most immediate clinical application, given their 48.5% baseline recurrence risk with conservative management. Guideline committees should incorporate cost-effectiveness frameworks, particularly WHO-recommended thresholds, when formulating resource-conscious recommendations for diverse healthcare settings. For shared decision-making with patients, utilizing the AAR of 36.4% rather than relative risk metrics provides more intuitive understanding of intervention benefits, as this translates directly to preventing one recurrence per three patients treated. Health systems must concurrently develop clinical pathways enabling timely VATS access presentation, as delays increase recurrence-associated costs [15]. Future research should prospectively validate these findings across varied healthcare economies while exploring techniques like awake VATS that may enhance cost-effectiveness, alongside integrating patient-reported outcomes to refine utility estimations in this young population.

## Conclusions

VATS significantly reduces recurrence risk (83.4% hazard reduction; ARR = 36.4%) versus chest tube drainage in first-episode PSP with blebs, with a median recurrence-free interval > 48 months versus 24 months. Its ICER (¥160,300 / QALY) falls below China’s WHO-recommended WTP threshold (¥287,247 / QALY), supported by a 99.14% cost-effectiveness probability. These findings challenge current guidelines favoring conservative initial management and advocate for early VATS in high-risk patients with blebs. Guideline committees should integrate cost-effectiveness frameworks and WHO thresholds to promote value-based care. Future research should validate these outcomes prospectively and explore awake VATS to enhance accessibility. 

## Supplementary Material

Supplementary Material 1: The flow chart of the study procedures is provided in Appendix 1 (Appendix 1. Flow chart of study procedures.drawio.pdf). The PSM algorithm and baseline characteristics before and after matching are detailed in Appendix 2 (Appendix 2. PSM algorithm and baseline characteristics before and after matching.xlsx). The economic evaluation (cost-effectiveness analysis) is presented in Appendix 3 (Appendix 3. Cost-effectiveness analysis.xlsx), which includes: Monte Carlo simulations, Cost-Effectiveness Plane, Cost-Effectiveness Acceptability Curve (CEAC), and Sensitivity Analysis.

## Data Availability

The datasets used and/or analyzed during the current study are available from the corresponding author on reasonable request.

## Abbreviations

ARR: Absolute Risk Reduction
CI: Confidence Interval
COPD: Chronic Obstructive Pulmonary Disease
CT: Computed Tomography
GDP: Gross Domestic Product
HR: Hazard Ratio
IBM: International Business Machines
ICER: Incremental Cost-Effectiveness Ratio
IQR: Interquartile Range
NNT: Number Needed to Treat
PAL: Prolonged Air Leak
PSM: Propensity Score Matching
PSP: Primary Spontaneous Pneumothorax
QALY: Quality-Adjusted Life-Year
SMD: Standardized Mean Difference
VATS: Video-Assisted Thoracoscopic Surgery
WHO: World Health Organization
WTP: Willingness-to-Pay
