# Supplementary figures and images for: Efficacy and cost-effectiveness of VATS versus chest tube drainage in first-episode primary spontaneous pneumothorax with blebs: a propensity score-matched retrospective study
Source: BMC Pulm Med. 2026 Feb 5;26:104. doi: 10.1186/s12890-026-04155-9 (PMC12964978; doi:10.1186/s12890-026-04155-9)

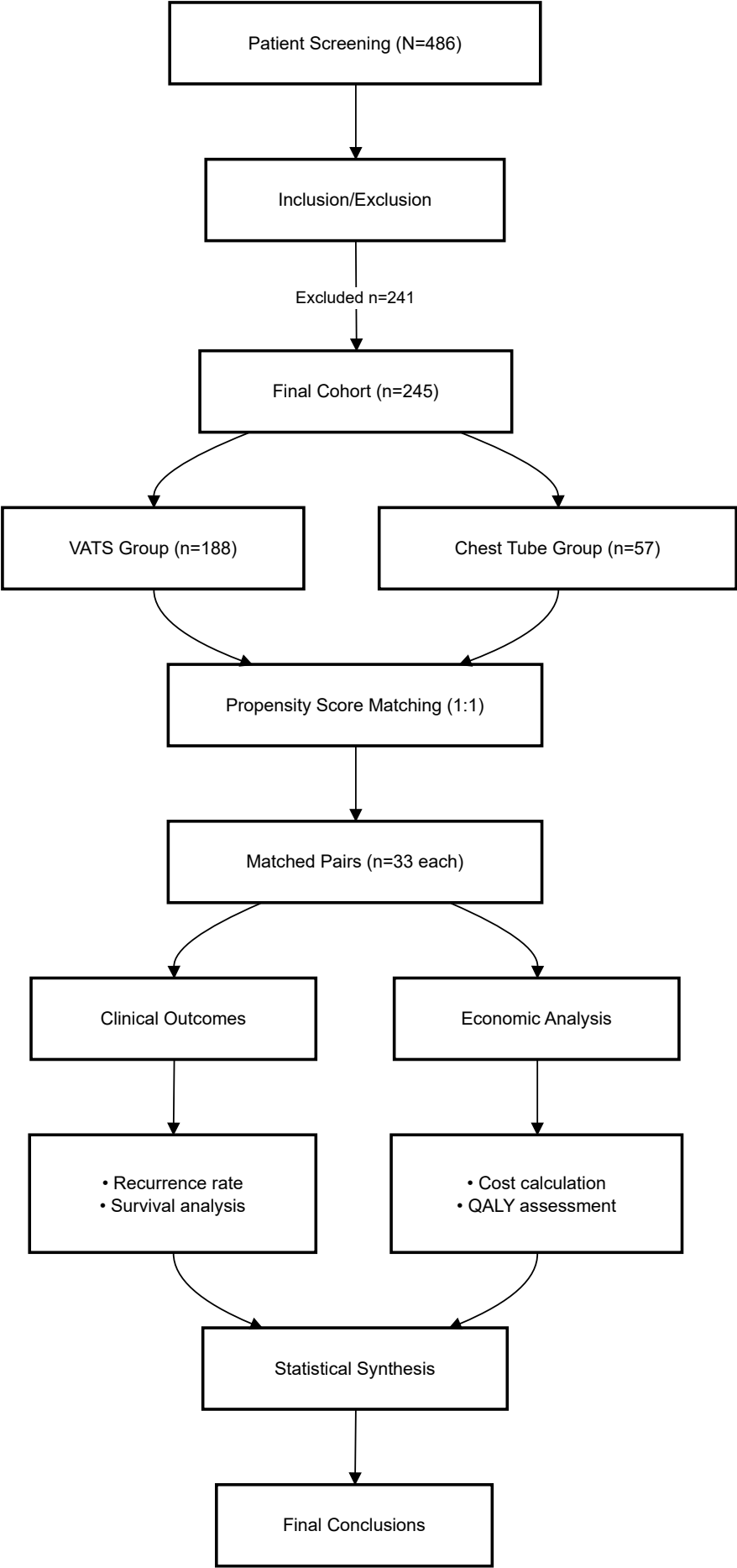

Supplement: Supplementary file 1 — Supplementary Material 1: The flow chart of the study procedures is provided in Appendix 1 (Appendix 1. Flow chart of study procedures.drawio.pdf). The PSM algorithm and baseline characteristics before and after matching are detailed in Appendix 2 (Appendix 2. PSM algorithm and baseline characteristics before and after matching.xlsx). The economic evaluation (cost-effectiveness analysis) is presented in Appendix 3 (Appendix 3. Cost-effectiveness analysis.xlsx), which includes: Monte Carlo simulations, Cost-Effectiveness Plane, Cost-Effectiveness Acceptability Curve (CEAC), and Sensitivity Analysis. [file 12890_2026_4155_MOESM1_ESM.zip › 12890_2026_4155_MOESM1_ESM.pdf]
